# A Novel Robot Visual Homing Method Based on SIFT Features

**DOI:** 10.3390/s151026063

**Published:** 2015-10-14

**Authors:** Qidan Zhu, Chuanjia Liu, Chengtao Cai

**Affiliations:** College of Automation, Harbin Engineering University, Harbin 150001, China; E-Mails: zhuqidan@hrbeu.edu.cn (Q.Z.); caichengtao@hrbeu.edu.cn (C.C.)

**Keywords:** visual homing, mismatching elimination, robot navigation, dynamic environment, catadioptric panoramic image

## Abstract

Warping is an effective visual homing method for robot local navigation. However, the performance of the warping method can be greatly influenced by the changes of the environment in a real scene, thus resulting in lower accuracy. In order to solve the above problem and to get higher homing precision, a novel robot visual homing algorithm is proposed by combining SIFT (scale-invariant feature transform) features with the warping method. The algorithm is novel in using SIFT features as landmarks instead of the pixels in the horizon region of the panoramic image. In addition, to further improve the matching accuracy of landmarks in the homing algorithm, a novel mismatching elimination algorithm, based on the distribution characteristics of landmarks in the catadioptric panoramic image, is proposed. Experiments on image databases and on a real scene confirm the effectiveness of the proposed method.

## 1. Introduction

The local navigation methods based on visual information (known as local visual homing) have attracted much attention in the mobile robot field [1,2,3]. Inspired by biological navigation, local visual homing provides the ability to guide the robot to return to a goal position only by relying on visual information [4,5,6]. It can calculate a homing direction by comparing the differences between the current image and the goal image, and in that homing direction, the robot can move from the current position to the goal position [7,8,9,10]. Note that neither the current image nor the goal image directly provide the depth information of the environment. By combining with the environmental topological map, the local visual homing algorithm can divide the complex large-scale navigation problem into a series of local navigation problems, which are easier to solve [11,12]. In the topological framework, the algorithm is used to solve navigation problems between adjacent connected nodes. Most previous research on visual homing was carried out under the assumption that environments are static. However, changes of the environment (objects, illumination) often happen in a real scene. Despite recent advances, the tolerance against changes in the environment is still widely unresolved [12,13]. So far, most studies of visual homing are limited to indoor environments. If the problem mentioned above can be resolved effectively, it will be beneficial to implement visual homing in more challenging outdoor environments [14,15].

Most local visual homing algorithms can be divided into the following four categories [16,17,18]. The first class is correspondence-based homing methods. They use feature extraction and matching to set up a set of correspondence vectors between the current image and the goal image. These vectors can be transformed into movement directions, and an overall homing vector can be obtained by combining these movement directions [19,20,21,22]. The second class is the DID (descent in image distances) methods. In these methods, the homing can be achieved by calculating the gradient descent direction in the image distance between the current image and the goal image [14,15,23,24]. The third class is the method based on the ALV (average landmark vector) model. ALV is a unit representation vector of a certain position. The homing direction can be acquired by the subtraction of ALV between the current position and the goal position [25,26,27]. The last class is a robust method, which is known as warping, despite the large amount of calculation. This method supposes that the robot performs virtual movement at the goal position according to the three motion parameters. These parameters separately describe the direction, distance and rotation of the robot movement from the goal position to its current position. The goal image is distorted according to the motion parameters, after which it is compared to the current image. The optimal parameter set can be found when the differences between the two images are minimal. The homing vector can be yielded by the optimal parameter set [13,28,29,30].

In the above four classes of visual homing methods, DID and ALV fail to reach the performance of the warping method [13,20]. Although the homing performance of some correspondence-based methods is superior, most of them need an external compass to adjust the current image and goal image to the same horizontal orientation in order to compute the homing vector [30]. However, warping does not need an external compass to align the orientations between the two images, as the change of orientation is one of the search parameters in warping. In conclusion, warping is an attractive visual homing method. Despite the above advantages, some problems of warping still remain to be solved. Firstly, the homing performance of warping is influenced greatly by the change of environment (objects, illumination), as it determines the homing vector according to the differences of the gray value between corresponding pixels of the horizon region in the current image and the goal image. Secondly, it is difficult to balance the homing accuracy and the amount of calculation. The homing accuracy of the warping method partly depends on the search interval of the parameter space. The smaller the search interval is, the higher the homing accuracy is. However, at the same time, the computation will also increase significantly. Although Möller *et al.* have improved the warping method, extending it to operate directly on two-dimensional images [30] and relaxing the equal distance assumption [13], there is no effective solution to the above two problems.

Motivated by the above problems, we propose a novel visual homing algorithm by combining the SIFT (scale-invariant feature transform) features with the warping method. Compared with the warping method, the proposed homing algorithm uses the SIFT features instead of the pixels of the horizon region in warping as landmarks, and its advantages can be stated as follows: (1) by using the good stability of SIFT features in translation, rotation, scaling, occlusion and illumination [31], the novel algorithm is more robust to the influence caused by the changes of the environment and has a better homing accuracy; (2) the proposed homing algorithm can resolve the conflict between the homing accuracy and the amount of calculation, because it can obtain the homing vector by solving a system of ternary equations in place of doing the exhaustive search in the parameter space. In addition, unlike most visual homing algorithms that need to unwarp the initial panoramic images, the proposed homing algorithm directly operates on the initial catadioptric panoramic images. As a result, the additional amount of calculation is reduced. It is according to the angle change of landmarks between the current image and the goal image that our homing algorithm computes the homing direction, so the matching accuracy of landmarks is crucial. Because of the complex imaging relations of catadioptric panoramic images, most existing mismatching elimination algorithms cannot directly operate on the initial panoramic images [32,33,34]. For the above reasons, in order to further improve the matching accuracy of landmarks, a novel mismatching elimination algorithm is proposed on the basis of the distribution characteristics of landmarks in the catadioptric panoramic image.

The remainder of the paper is organized as follows: Section 2 describes the design of the proposed homing algorithm. The selection of landmarks and the design of the proposed mismatching elimination algorithm are introduced in Section 3. Section 3 also shows the overview of the proposed homing method. The results and analysis of the experiments are mainly presented in Section 4. Section 5 draws conclusions and points out the focus of future research.

## 2. Homing Algorithm

For the derivation of the warping method, refer to [28]. Similar to the warping method, our homing algorithm is also based on the equal distance assumption, which assumes that all of the surrounding landmarks have identical distances from the goal position. Although this assumption is usually violated, Franz *et al.* have proven that the error due to this assumption decreases when the robot approaches the goal [28].

**Figure 1:**
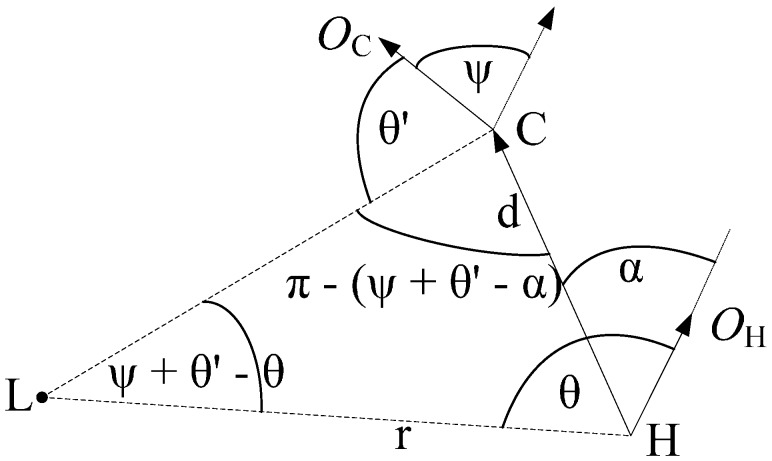
Derivation of the proposed homing algorithm.

The geometric relations of the homing algorithm are shown in Figure 1. The goal position and the current position of the robot are indicated separately by H and C. L is a landmark in the scene. The distance from L and H is denoted by r. The initial orientation of the robot at position H is shown by *O*_H_. The robot moves away from H in direction *α* relative to its initial orientation *O*_H_ and covers a distance d to C. After the movement, the initial orientation changes by *ψ*. The new orientation of the robot is shown by *O*_C_. The dashed arrow at position C indicates the old orientation. In order to facilitate the derivation, we assume that the landmark L corresponds to some feature in the real scene. The angle between landmark L and orientation *O*_H_ is denoted by *θ*. As the robot moves, the angle *θ* changes to *θ*′, that is the angle between landmark L and new orientation *O*_C_. Applying the law of sines to the triangle LHC, we can obtain the following equation:

(1) $$\frac{\sin\left(\psi +\theta^{\prime }-\theta \right)}{d}=\frac{\sin\left(\pi -[\psi +\theta^{\prime }-\alpha ]\right)}{r}$$

Equation (1) can be rearranged as follows:

(2) sin(*ψ* + *θ*′ − *θ*) = *ρ*sin(*ψ* + *θ*′ − *α*)

where *ρ* = *d*/*r*. There are three unknown parameters (*ρ*, *ψ*, *α*) in Equation (2). *ψ* and *α* are invariable for all of the landmarks in a certain scene. Suppose there are three landmarks L1, L2 and L3 in the scene. The angles between these landmarks and the orientation of the robot are separately denoted by *θ*_1_, *θ*_2_ and *θ*_3_ in the goal image. In the current image, the corresponding angles are denoted by *θ*′_1_, *θ*′_2_ and *θ*′_3_. Substitute (*θ*_1_, *θ*′_1_), (*θ*_2_, *θ*′_2_) and (*θ*_3_, *θ*′_3_) into Equation (2), and we can obtain the following equations:

(3) $$\left\{ \begin{array}{l} \sin\left({\theta^{\prime }}_{1}-\theta_{1}+\psi \right)=\rho_{1}\sin\left({\theta^{\prime }}_{1}-\alpha +\psi \right)\\ \sin\left({\theta^{\prime }}_{2}-\theta_{2}+\psi \right)=\rho_{2}\sin\left({\theta^{\prime }}_{2}-\alpha +\psi \right)\\ \sin\left({\theta^{\prime }}_{3}-\theta_{3}+\psi \right)=\rho_{3}\sin\left({\theta^{\prime }}_{3}-\alpha +\psi \right) \end{array}\right.$$

Before Equation (3) can be applied for homing, two problems have to be explained:

1. The distance d is identical for all of the landmarks in the scene when the robot arrives at current position C from goal position H. According to the equal distance assumption, the distance r is also identical for all of the landmarks. Based on the above two conditions, there exist the relation *ρ*_1_ = *ρ*_2_ = *ρ*_3_.

2. The angles *θ*_1_, *θ*_2_, *θ*_3_ and *θ*′_1_, *θ*′_2_, *θ*′_3_ can be worked out if the positions of landmarks L1, L2 and L3 in the panoramic image are known.

By solving Equations (3), we can get a parameter set (*ρ*, *ψ*, *α*). The homing direction *β* relative to the new orientation of the robot can be computed as follows:

(4) *β* = *π* + *α* − *ψ*

It can be seen from Equations (3) to (4) that the homing direction can be determined by only three landmarks. As the number of landmarks is usually more than three in the real scene, a number of parameter sets, such as (*ρ*_1_, *ψ*_1_, *α*_1_), (*ρ*_2_, *ψ*_2_, *α*_2_)…(*ρ*_n_, *ψ*_n_, *α*_n_), can be obtained by solving Equation (3). In order to get the optimal parameter $\hat{\alpha }$, we first give the definition of the sum of squares of deviations:

(5) $$\alpha_{SSD}=\sum_{i=1}^{n}diff{\left(\alpha_{i}-\bar{\alpha }\right)}^{2}$$

where *n* is the number of parameter sets, $\bar{\alpha }$ changes from −*π* to *π* and *diff* (*αi* − $\bar{\alpha }$) yields the difference between two angles, which is defined as:

(6) $$diff\left(\alpha_{i}-\bar{\alpha }\right)=\left\{ \begin{array}{ll} 2\pi -\left|\alpha_{i}-\bar{\alpha }\right|, & \left|\alpha_{i}-\bar{\alpha }\right|\geq \pi \\ \left|\alpha_{i}-\bar{\alpha }\right|, & \left|\alpha_{i}-\bar{\alpha }\right|<\pi \end{array}\right.$$

According to Equation (5), we acquire $\hat{\alpha }={\bar{\alpha }}_{SSD\min}$, and ${\bar{\alpha }}_{SSD\min}$ is the value of $\bar{\alpha }$ when *α_SSD_* is minimum. The calculation of $\hat{\psi }$ is the same as that of $\hat{\alpha }$. The final homing direction $\hat{\beta }$ can be determined as follows:

(7) $$\hat{\beta }=\pi +\hat{\alpha }-\hat{\psi }$$

## 3. Landmark Optimization and Overview of the Proposed Method

For the purpose of guiding the robot to return to the goal position, all of the visual homing algorithms need to acquire accurate and reliable information from the image. It can be seen from Section 2 that the calculation precision of the proposed homing algorithm mainly depends on the landmarks extracted from the image, so both the selection of reliable landmarks and the matching accuracy are crucial to the homing performance.

### 3.1. Landmark Selection

In this section, we will make the qualitative analysis of the landmark selection. Gillner *et al.* [35] suggested the principle of landmark selection, which includes the following three points: (1) uniqueness: landmarks in the scene must be unique and can be identified clearly; (2) reliability: the reliability of landmarks refers to the stable visibility, which means that the landmarks should be always detected every time the robot travels to the same position; and (3) relevance: the relevance of landmarks is defined as their importance in determining the homing direction.

According to the above three principles, we choose SIFT features in the image as landmarks. Firstly, as SIFT features are highly distinctive and can be correctly matched with high probability against large features [31], they can meet the uniqueness condition of landmarks. Secondly, as SIFT features are invariant to image translation, rotation, scale and are stable with respect to the changes in illumination [31], the reliability condition can be satisfied. At last, as the angles between SIFT features and the orientations of the robot can be computed by the localization of SIFT features, we can get the homing direction by utilizing Equations (3)–(7). The relevance condition can be satisfied.

### 3.2. Mismatching Elimination

In order to obtain a larger field of view and a faster processing speed, the proposed homing algorithm directly extracts SIFT features from the initial catadioptric panoramic images as landmarks. Although the SIFT features have good performance, there still exist some mismatching points in the real scene. According to Section 2, the mismatching of the landmarks can generate the wrong corresponding angle pair (*θ*, *θ*′), which will affect the homing precision and even cause the failure of homing. To solve the above problems, we present two constraints according to the distribution characteristics of landmarks in the initial panoramic images and propose a novel mismatching elimination algorithm based on these constraints.

#### 3.2.1. Two Distribution Constraints

**Figure 2:**
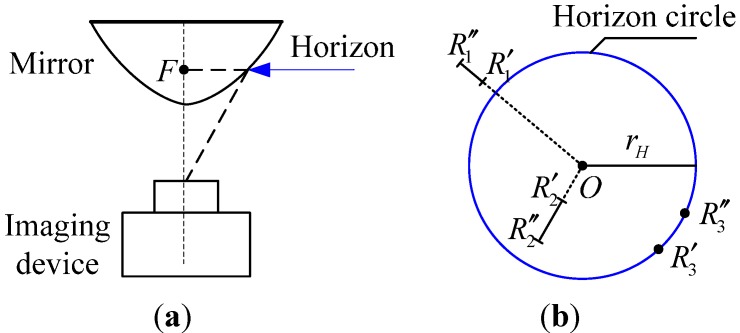
Distribution Constraint 1. (**a**) The formation of the horizon circle; (**b**) the distribution constraint of the landmarks based on the horizon circle.

The panoramic images used in this paper are all generated by the catadioptric panoramic imaging system based on the hyperbolic mirror. Before presenting the mismatching elimination algorithm, we firstly introduce two distribution constraints of the landmarks as follows:

Distribution Constraint 1: As shown in Figure 2a, in the catadioptric panoramic imaging system based on the hyperbolic mirror, a curved mirror is used to form the image of the surroundings. Under the curved mirror is located the camera, whose optic axis points to the mirror. The curved mirror works together with the camera to collect the panoramic image. Ideally, in the initial panoramic image, there exists a circle whose center is just at the center of the image. For landmarks located at the same horizontal level with focus F of the curved mirror, their projection must be on that circle. As shown in Figure 2b, we call this circle the horizon circle in this paper. According to the characteristics of the catadioptric panoramic imaging system, suppose the robot moves on a plane; the landmarks in the scene can be divided into three categories: (1) the landmarks are located higher than focus F; their imaging radius range is always outside the horizon circle, shown as *R*′_1_*R*″_1_; (2) the landmarks are located lower than focus F; their imaging radius range is always within the horizon circle, shown as *R*′_2_*R*″_2_; and (3) the landmarks are located at the same horizontal level with focus F. As *R*′_3_(*R*″_3_) shows, the imaging radius range of these landmarks is always on the circle as the robot travels. In this paper, we employ *γ_IO_*(*L*) to determine the position relationship between the landmark L and the horizon circle in quantization, which can be defined as follows:

(8) $$\gamma_{IO}\left(L\right)=\left\{ \begin{array}{ll} -1, & r_{L}<r_{H}\\ 0, & r_{L}=r_{H}\\ 1, & r_{L}>r_{H} \end{array}\right.$$

where *r_L_* is the imaging radius of the landmark and *r_H_* is the radius of the horizon circle.

According to the above principle, suppose *L_T_* is a landmark to be tested in the goal image, and ${\tilde{L}}_{T}$ denotes its matching landmark in the current image. If the pair of landmarks (*L_T_*, ${\tilde{L}}_{T}$) matches correctly, there will exist the relation *γ_IO_*(*L_T_*) = *γ_IO_*(${\tilde{L}}_{T}$). In other words, if *γ_IO_*(*L_T_*) ≠ *γ_IO_*(${\tilde{L}}_{T}$), (*L_T_*, ${\tilde{L}}_{T}$) is a pair of mismatching landmarks.

**Figure 3:**
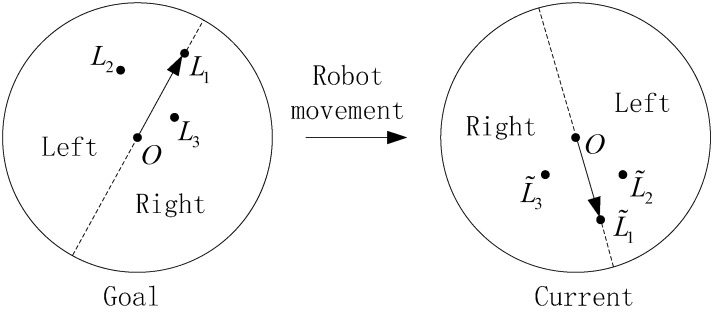
Distribution Constraint 2.

Distribution Constraint 2: As shown in Figure 3, the left picture shows the goal image, and the right picture shows the current image. *O* is the center of the image. *L*_1_, *L*_2_ and *L*_3_ are three landmarks in the goal image. ${\tilde{L}}_{1}$, ${\tilde{L}}_{2}$ and ${\tilde{L}}_{3}$ are their matching landmarks in the current image, respectively. (*L*_1_, ${\tilde{L}}_{1}$) is a pair of landmarks to be tested. (*L*_2_, ${\tilde{L}}_{2}$) and (*L*_3_, ${\tilde{L}}_{3}$) are used as reference landmark pairs. The two directed lines radiate from the center *O* and point to *L*_1_ and ${\tilde{L}}_{1}$, based on which both the goal image and the current image can be divided into the left half plane and the right half plane. As the robot moves from the goal position to the current position, the reference landmark pairs (*L*_2_, ${\tilde{L}}_{2}$) and (*L*_3_, ${\tilde{L}}_{3}$) will separately appear in the same half plane of the two images on the condition that (*L*_1_, ${\tilde{L}}_{1}$) are matching correctly. Conversely, if (*L*_1_, ${\tilde{L}}_{1}$) is a pair of mismatching landmarks, (*L*_2_, ${\tilde{L}}_{2}$) and (*L*_3_, ${\tilde{L}}_{3}$) may randomly appear in any position of the two images.

Suppose the coordinates of *O*, *L*_1_ and *L*_2_ are (*x_O_*,*y_O_*), (*x_T_*,*y_T_*) and (*x_R_*,*y_R_*), respectively. The equation of the directed line *OL*_1_ is *Ax* + *By* + *C* = 0, where *A* = *y_T_* − *y_O_*, *B* = *x_O_* − *x_T_* and *C* = *x_T_y_O_* − *x_O_y_T_*. The positional relation of the reference landmark *L*_2_ can be determined as follows:

(9) *D_R_* = *Ax_R_* + *By_R_* + *C*

In Equation (9): (1) if *D_R_* < 0, *L*_2_ is located in the left half plane; (2) if *D_R_* > 0, *L*_2_ appears in the right half plane; (3) if *D_R_* = 0, *L*_2_ is on the line *OL*_1_. We employ *γ_LR_*(*L*) to indicate the positional relation of a reference landmark in quantization, which is defined as:

(10) $$\gamma_{LR}\left(L\right)=\left\{ \begin{array}{ll} -1, & D_{R}<0\\ 0, & D_{R}=0\\ 1, & D_{R}>0 \end{array}\right.$$

#### 3.2.2. Mismatching Elimination Algorithm

Suppose *S*_1_ is the initial set of matching landmarks, which are extracted from the goal image *I_H_* and the current image *I_C_*. The matching landmark pair (*L_T_*, ${\tilde{L}}_{T}$) ∈ *S*_1_ is the one to be tested, where *L_T_* and ${\tilde{L}}_{T}$ are landmarks extracted separately from *I_H_* and *I_C_*. The proposed mismatching elimination algorithm mainly includes two phases, which can be presented as follows:

**Figure 4:**
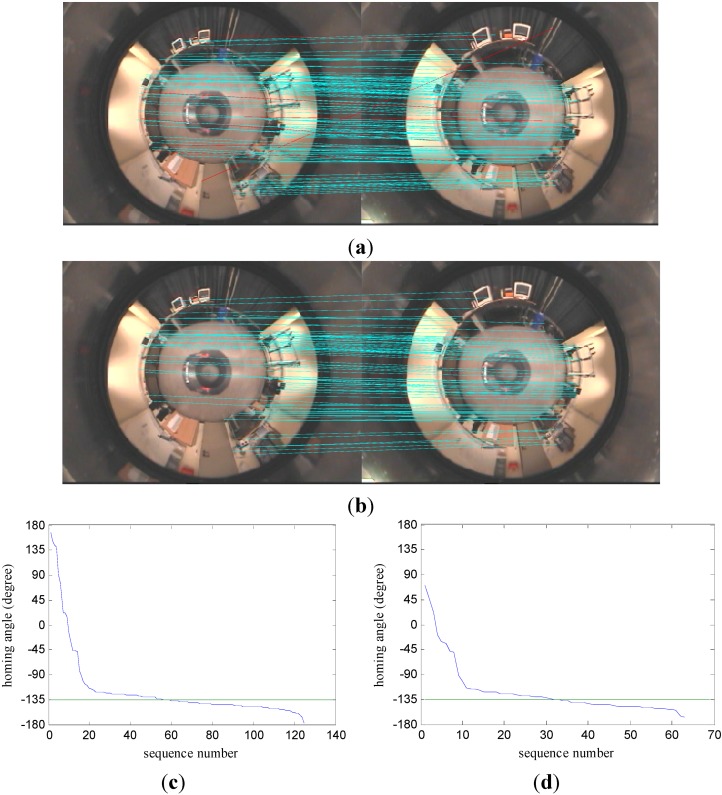
The performance of the proposed mismatching elimination algorithm. (**a**) The matching of landmarks before mismatching elimination; (**b**) the matching of landmarks after mismatching elimination; (**c**,**d**) the distribution of homing angles computed by the proposed homing algorithm before and after mismatching elimination.

The first phase: According to Constraint 1, we firstly calculate *γ_IO_*(*L_T_*) and *γ_IO_*(${\tilde{L}}_{T}$) for each pair (*L_T_*, ${\tilde{L}}_{T}$) ∈ *S_1_*. If *γ_IO_*(*L_T_*) ≠ *γ_IO_*(${\tilde{L}}_{T}$), the corresponding landmark pair will be discarded from set *S*_1_; if *γ_IO_*(*L_T_*) = *γ_IO_*(${\tilde{L}}_{T}$), the corresponding landmark pair will be reserved. After the preliminary filtration of set *S*_1_, the remaining matching landmark pairs make up set *S*_2_.

The second phase: For each matching landmark pair (*L_T_*, ${\tilde{L}}_{T}$) ∈ *S*_2_, we firstly calculate the distance between *L_T_* and other landmarks in *I_H_* and choose *n_R_* landmarks that are nearest to *L_T_* as reference landmarks to constitute set *S_RH_*, then find their matching landmarks in *I_C_* to constitute set *S_RC_*. The correctness of each matching landmark pair to be examined can be evaluated by the reference landmark pairs in set *S_RH_* and set *S_RC_*, and the result will be registered in *V_m_*, whose initial value is zero. According to Constraint 2, we separately calculate *γ_LR_*(*L_R_*) and *γ_LR_*(${\tilde{L}}_{R}$) for each reference landmark *L_R_* ∈ *S_RH_* and its matching landmark ${\tilde{L}}_{R}$ ∈ *S_RC_*. If *γ_LR_*(*L_R_*) = *γ_LR_*(${\tilde{L}}_{R}$), the current reference landmark pair supports the correctness of the matching pair to be examined, and the value of *V_m_* increases by one; if *γ_LR_*(*L_R_*) ≠ *γ_LR_*(${\tilde{L}}_{R}$), the matching pair to be examined is regarded as the wrong one, and the value of *V_m_* does not change. Suppose the threshold is *V_TH_*; a matching pair *m* = (*L_T_*,${\tilde{L}}_{T}$) ∈ *S*_2_ can be directly judged by the value of its *V_m_*. If *V_m_* ≥ *V_TH_*, *m* is a correct matching pair and needs to be reserved; if *V_m_* < *V_TH_*, *m* is regarded as a mismatching pair and will be removed from set *S*_2_. The remaining matching pairs will be used as the final landmarks and constitute set *S*_3_.

In order to evaluate the performance of the proposed mismatching elimination algorithm, the panoramic image databases provided by Bielefeld University [19] are adopted. The image databases will also be used in the subsequent homing experiments, and the details will be introduced in Section 4.1. Figure 4 shows the performance of the proposed mismatching elimination algorithm. In Figure 4a, the red lines indicate the obvious mismatching landmarks. It can be seen from Figure 4b that these mismatching pairs are eliminated effectively by the proposed algorithm. In Figure 4c,d, green lines denote the ideal homing angle. After eliminating the mismatching landmarks, the distribution of the homing angles calculated by the proposed homing algorithm is much closer to the ideal homing angle, as shown in Figure 4c,d. Experimental results show that the proposed algorithm in this section can effectively eliminate the mismatching landmark pairs and further improve the computational accuracy of the proposed homing algorithm.

### 3.3. Overview of the Proposed Visual Homing Method

**Figure 5:**
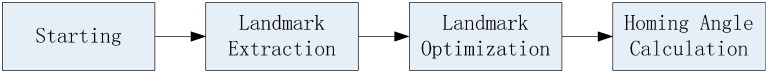
Flow diagram of the proposed homing method.

As shown in Figure 5, the procedure of our homing method mainly includes three steps as follows:

(1) Landmark extraction: We extract SIFT features from the goal image *I_H_* and the current image *I_C_*, then match the features to form the initial landmark set *S*_1_.

(2) Landmark optimization: The primary purpose of this step is to eliminate the mismatching landmark pairs in set *S*_1_. The proposed algorithm in this paper solves this problem in a coarse-to-fine hierarchical way. First of all, according to the landmark distribution Constraint 1, the matching landmark pairs in set *S*_1_ are filtered preliminarily by making full use of the distribution characteristic between the landmarks and the horizon circle. The remainder in set *S*_1_ form set *S*_2_. After that, with the help of the landmark distribution Constraint 2, the mismatching pairs in set *S*_2_ are further removed on the basis of the relative distribution relations of the landmarks in an initial panoramic image. The final landmark set *S*_3_ consists of the remaining landmark pairs in set *S*_2_.

(3) Homing angle calculation: According to Section 2, we can get an angle pair (*θ*, *θ*′) for each pair (*L*,$\tilde{L}$) ∈ S3. The final homing direction $\hat{\beta }$ can be worked out based on Equations (3) to (7).

## 4. Experiments

### 4.1. Image Databases and Robot Platform

**Figure 6:**
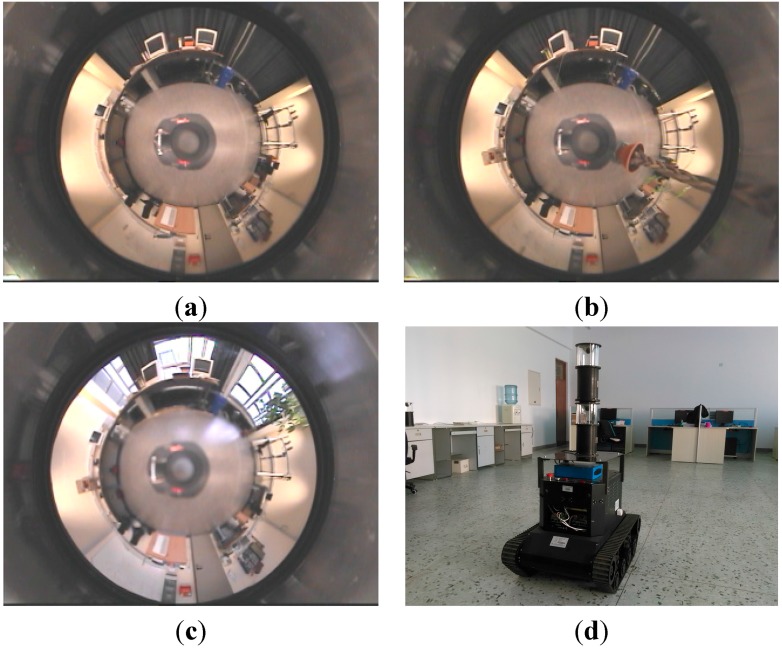
Panoramic sample images and robot platform. (**a**–**c**) The samples of three image databases: original, arboreal and day; (**d**) the robot platform for experiments in the real scene.

The panoramic image databases used in this paper were collected by the Computer Department of Bielefeld University. These databases were widely applied to the tests of robot visual homing algorithms. The panoramic images in the databases were all collected by the catadioptric imaging system in different scenes. The capture grid of the above image databases measured 2.7 × 4.8 m, and images were collected uniformly spaced 0.3-m apart. The resolution of these images is 752 × 564. The images acquired in three different scenes of original, arboreal and day were used for the experiments. Three panoramic sample images are shown in Figure 6a to c, and the corresponding scene conditions can be introduced as follows: (a) original means a common room with doors and windows shut, and its overhead fluorescent light bars were on; (b) arboreal represents a tall plant that was added in the capture grid; and (c) day means images that were collected with curtains open in full daylight. The databases and their further details can be accessed through [36].

In this paper, a tracked mobile robot was used in the experiments in the real scene, as shown in Figure 6d. The panoramic imaging system mounted on the top of the robot was composed of a hyperbolic mirror and a camera with a resolution of 1024 × 768. Although there were two sets of panoramic imaging equipment on the robot, we just used the one below in the experiments. Image processing and robot movement were controlled by an onboard computer (Pentium (R), 2 GHz).

### 4.2. Parameter Settings for Experiments

The parameter settings in the experiments are shown in Table 1. In this paper, we employ the SIFT features of the image as landmarks. On the premise of accurate results, we modified several parameters recommended by Lowe in [31] to get more SIFT features. Firstly, the number of scale layers *S* where SIFT features are extracted is increased from 3 to 5, which can both increase the total number of extracted landmarks and maintain a reasonable time of execution. Secondly, the response threshold of extreme points *T_DOG_* in the difference of Gaussian images is decreased from 0.08/*S* to 0.04/*S* in order to get more features from areas of low contrast, as indoor environments often contain such areas.

**Table 1:** Parameters for the experiments.

Parameters	Value	Parameters	Value
*S*	5	*α_W_*	[0, 355]/72/5
*T_DOG_*	0.04/*S*	*n_R_*	5
*ρ_W_*	[0, 0.95]/20/0.05	*V_TH_*	4
*ψ_W_*	[0, 355]/72/5		

In Table 1, the parameter format of *ρ_W_*, *ψ_W_* and *α_W_* is shown as search range/search steps/resolution. In order to give full play to the performance of the warping method in the experiments, we adopted the parameters recommended by Möller in [30]. The search range of *ρ_W_* is [0, 0.95], and there are 20 search steps. The settings of *ψ_W_* and *α_W_* are the same, whose search range is [0, 355], and there are 72 search steps. In addition, according to the requirements of the proposed homing algorithm, the number of the reference landmark pairs *n_R_* and the threshold *V_TH_* for eliminating the mismatching landmarks were separately set to 5 and 4.

We separately took 100 pairs of images from the three image databases randomly. These image pairs were used as the goal image and the current image. Based on the parameter settings in Table 1, we determined the average computation time for two methods (2 GHz Pentium (R), MATLAB R2007b), as shown in Table 2. It can be seen that the average computation time of the warping method for each homing test was about 40% more than that of our method. We consider the settings in Table 1 reasonable enough for the comparison of homing performance.

**Table 2:** Average computation time for the two methods.

Method	Average Computation Time (s)		
	*original*	*arboreal*	*day*
Warping	21.051	18.643	19.541
Proposed	14.559	12.638	13.854

### 4.3. Performance Metrics

According to the previous presentation, the goal position and the current position are denoted separately by H and C in the experiments. In this paper, three metrics known as angular error (AE), average homeward component (AHC) and return ratio (RR) are adopted to evaluate the performance of the proposed method.

For a pair of H and C, suppose the homing angle computed by the homing algorithm is indicated by *β*_homing_. *β*_ideal_ represents the ideal homing angle, which directly points from C to H. The angular error can be determined as:

(11) *AE*(H,C) = *diff*(*β*_homing_ − *β*_ideal_)

where the function of *diff*() refers to Equation (6).

The average homeward component, used in the homing experiments frequently, is the evaluation criterion, which can measure both the validity and the angular deviation of the computed homing angles. As long as the value of AHC always stays above zero, the robot travels nearer to the goal position; the closer the value of AHC is to 1, the closer the movement direction of the robot gets to the ideal homing direction. Based on the angular error, the average homeward component can be defined as follows:

(12) $$AHC\left(n\right)=\cos\left(\frac{1}{n}\sum_{i=1}^{n}AE\left(H_{i},C_{i}\right)\right)$$

where *n* indicates the number of different pairs (H, C) selected in the experiment scene. In the experiments of AHC, we separately took 100 pairs of images from the image databases randomly according to the distance between C and H, which ranges from 30 to 390 cm in steps of 30 cm.

The last performance metric is the return ratio [18,19], which is defined as the successful percentage of homing trials. The return ratio can be computed by carrying out simulated homing trials on the capture grid of the image databases. A dummy robot is placed at all integer grid positions and allowed to move according to the homing vectors, which have been pre-computed with the two methods. The robot moves at a step of *r*_h_, which is generally determined by the ratio between the actual step length of the robot and the sampling interval of the images. The value of *r*_h_ was set to 0.8 in the trials. *β*_h_(*x*,*y*) denotes the homing angle pre-computed at each position in the capture grid, and the motion direction of the robot is determined by *β*_h_(*x*,*y*), whose position is closest to the current position. A trial is considered successful if the robot can reach a place within a given distance threshold of H from C. The threshold was set to 0.5. The result of a homing trial at position C can be evaluated as follows:

Step 1: The robot moves a step according to the corresponding *β*_h_(*x*,*y*).
Step 2: If the following two cases happen, jump to Step 4.
  Case 1: The robot arrives at the goal position H.
  Case 2: The robot travels a distance longer than half of the perimeter of the capture grid.
Step 3: Continue to perform Step 1.
Step 4: If Case 1 happens, the homing trial is successful; if Case 1 does not happen and Case 2 happens, the trial has failed.

We define *λ*(H,C) as a binary evaluation function with a value of 1 for successful homing and 0 for unsuccessful homing. The return ratio is determined as:

(13) $$RR\left(H\right)=\frac{1}{n}\sum_{i=1}^{n}\lambda \left(H,C_{i}\right)$$

where *n* indicates the number of different current positions selected in the experiment scene.

### 4.4. Homing Experiments on Image Databases

The image databases introduced in Section 4.1 were used to perform the experiments. The experiment environment of the scene in this section was divided into two classes: (1) static environment: the surroundings of the scene remain static during the experiment; (2) dynamic environment: the objects or illumination in the scene changes during the experiment. According to different experiment environments, three different groups of experiments were conducted: (1) Group 1: the main goal is to test the homing performance of the proposed method under static conditions; both current images and goal images were selected from the database original, and this experiment condition was represented by original-original; (2) Group 2: the main goal is to test the homing performance of the proposed method when the objects in the scene change; the changing of objects was simulated by using cross-database experiments, *i.e.*, the current images were taken from the database original, and the goal images were taken from the database arboreal; the experiment condition was represented by original-arboreal; (3) Group 3: the main goal is to test the homing performance of the proposed method when the illumination of the scene changes; similar to the second group of experiments, the current images were taken from the database original and the goal images were taken from the database day, in order to simulate the changing of illumination; the experiment condition was represented by original-day.

**Figure 7:**
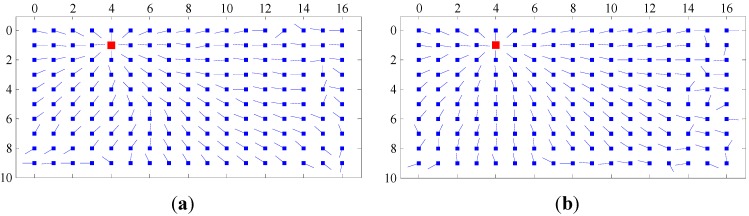

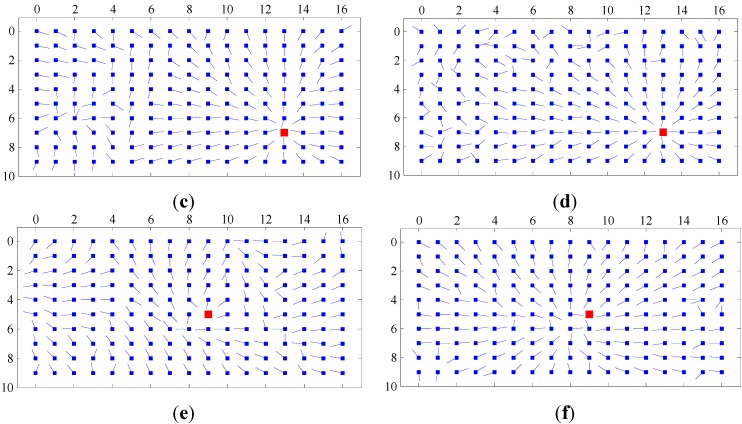
Homing vector fields. (**a**,**c**,**e**) The homing vectors generated by the warping method; (**b**,**d**,**f**) the homing vectors generated by the proposed homing method.

**Figure 8:**
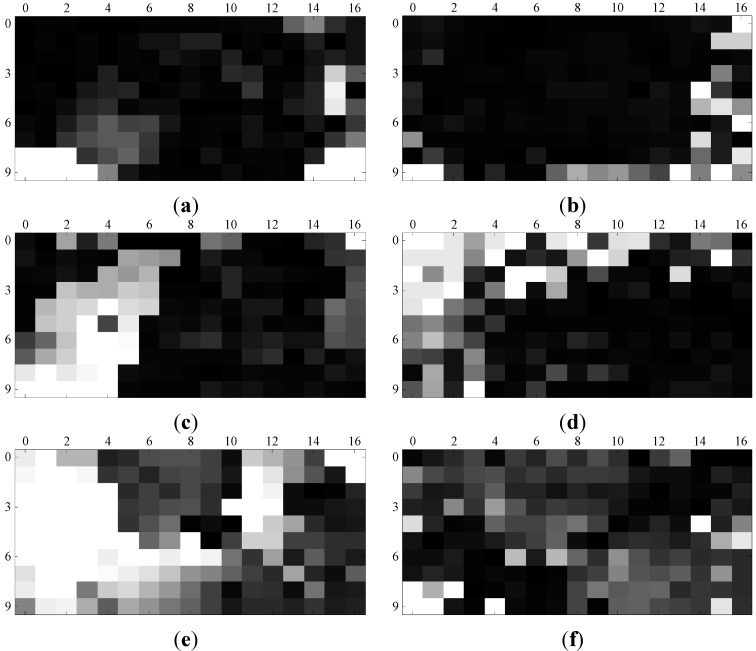
Angular error (AE) results. (**a**,**c**,**e**) The homing angular errors generated by warping method; (**b**,**d**,**f**) the homing angular errors generated by the proposed homing method.

Figure 7 shows the homing vector fields for the warping method and the proposed method. (1, 4), (7, 13) and (5, 9) in the capture grid were selected as goal positions, and the corresponding experiment conditions were original-original, original-arboreal and original-day, respectively. In Figure 7, the current positions are marked by blue squares, and the goal positions are marked by red squares. The homing direction is denoted by the line put out from the blue square. Figure 8 shows the AE results for two homing methods. The experiment conditions and goal positions are the same as the settings of Figure 7. The gray scale of each grid indicates the AE value of the corresponding (x, y) position in the capture grid of the database. Its changing from black to white represents the value ranging from 0 to the maximum of AE computed by the two methods. For observing, we change the threshold of white to 100 in the experiments. From Figure 7 to Figure 8, we can draw the following conclusions: Most of the homing directions computed by our method are more accurate than those computed by warping method. The AE of our homing method is lower than that of the warping method on the whole.

**Figure 9:**
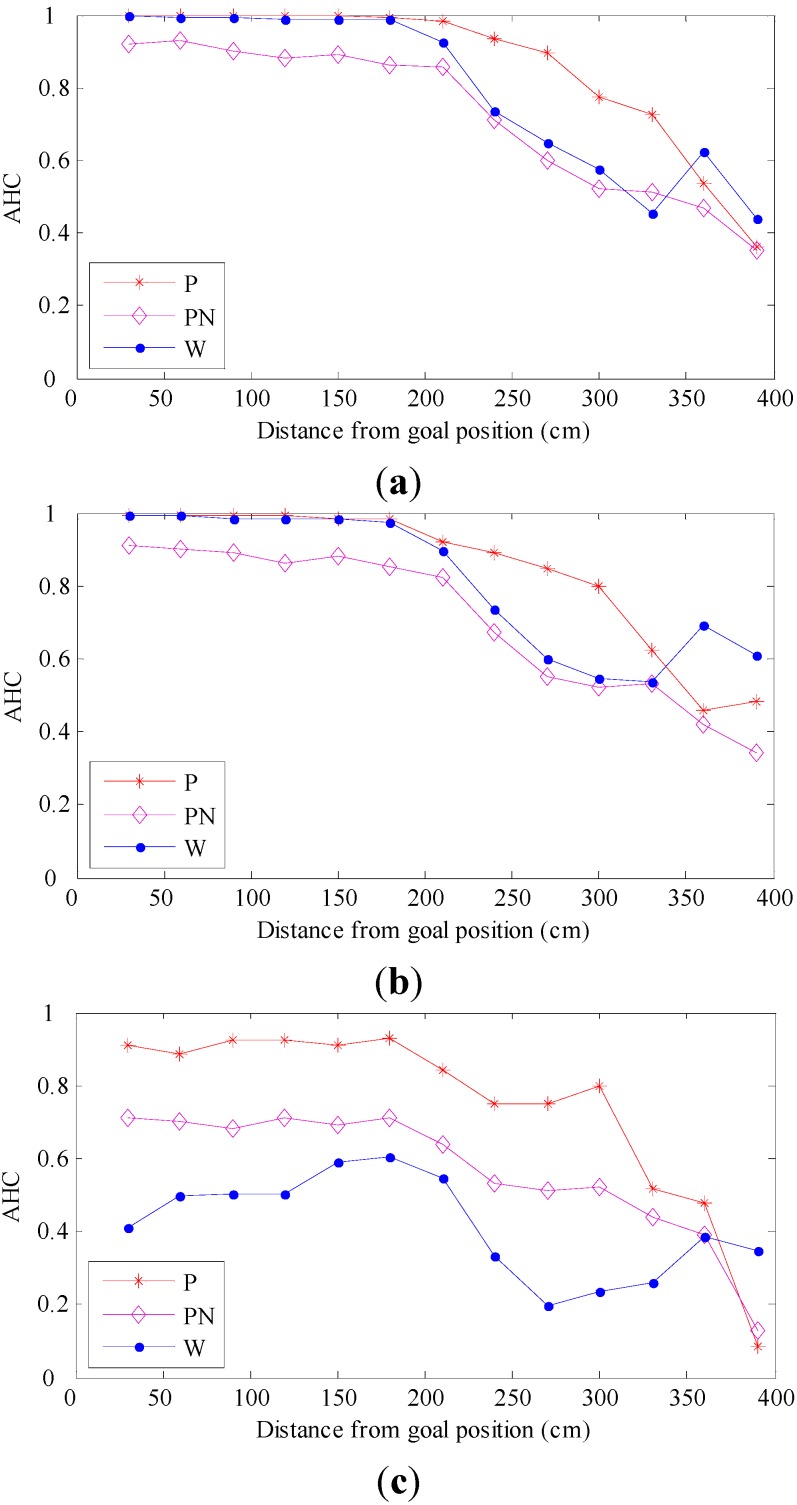
Average homeward component (AHC) results. (**a**–**c**) The distribution of AHC under the experiment conditions: original-original, original-arboreal and original-day. P, proposed homing method; PN, proposed homing method without a mismatching elimination step; W, warping method.

Figure 9 shows the distribution of AHC according to the distance between C and H. The corresponding experiment conditions for Figure 9a–c were original-original, original-arboreal and original-day, respectively. From Figure 9, it can be seen from the changing trends of “P” and “PN” that the mismatching elimination step can effectively improves the homing performance. It also can be seen intuitively from the changing trends of “P” and “W” that the AHC of our homing method is superior to that of the warping method on the whole. In the three groups of experiments, the AHC of two methods is always above 0, which indicates that both methods have the ability to guide the robot to approach the goal position gradually. From Figure 9a,b, although the change of a tall plant in the experiment scene leads to a slight decrease in AHC for two methods, our homing method still performs better. As shown in Figure 9a,c, when the illumination of the experiment scene changes, the performance of the warping method drops dramatically, while the performance of our homing method drops slightly. In conclusion, the results show that our homing method has better robustness to the changes of the environment. In Figure 9, we can see an interesting phenomenon: when the distance between C and H is approximately within the range 30 to 330 cm, the AHC of our method is closer to 1 than that of the warping method, which shows that the average AE of our homing method is smaller; when the distance between C and H is approximately within the range 360 to 390 cm, the AHC of our homing method is smaller than that of the warping method, which indicates that the average AE of our homing method is higher. The main reasons for this situation are as follows: (1) when the distance between C and H is short (30 to 330 cm), there are more correct matching landmarks due to minor differences between the two images; consequently, the proposed mismatching elimination algorithm can effectively remove the mismatching landmarks, and our homing algorithm can get higher calculation accuracy; (2) when the distance between C and H is far (360 to 390 cm), the number of matching landmarks is smaller, and among them, there are more mismatching landmarks. For this reason, the proposed algorithm cannot effectively eliminate the mismatching landmarks, which makes the calculation accuracy of our homing algorithm decrease significantly.

**Figure 10:**
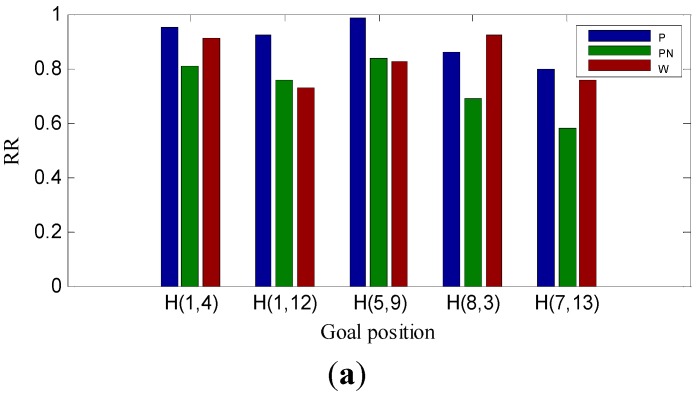

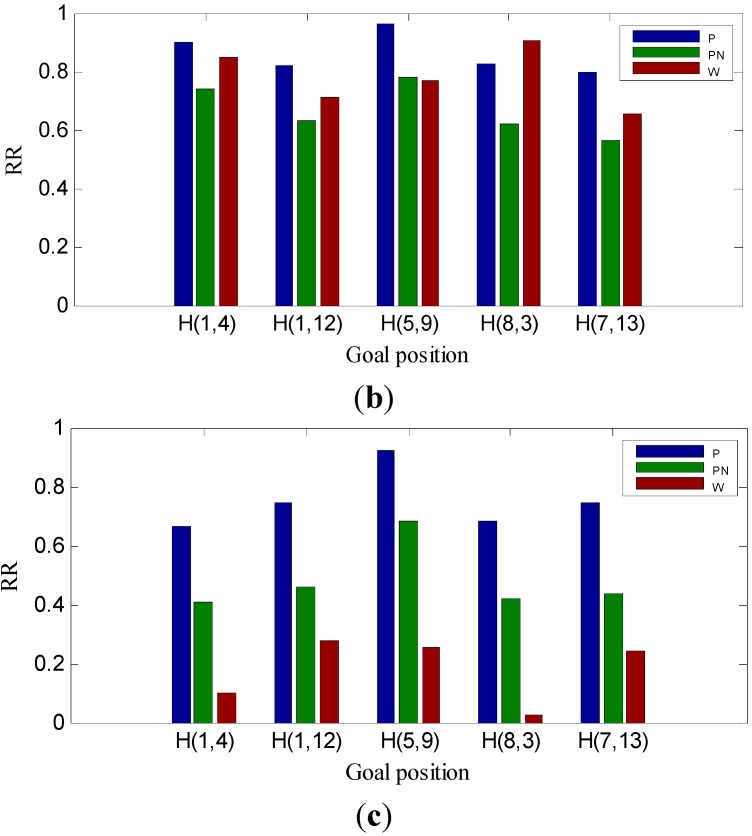
Return ratio (RR) results. (**a**–**c**) The RR for five goal positions under the experiment conditions: original-original, original-arboreal and original-day.

Figure 10 shows the RR for the two methods under the three experiment conditions. “P” represents the proposed homing method; “PN” represents the proposed homing method without mismatching elimination step; and “W” represents the warping method. Trying to avoid the influence of randomness, we chose (1, 4), (1, 12), (5, 9), (8, 3) and (7, 13) in the capture grid of the image databases as goal positions, which are uniformly distributed in the experiment scene. From Figure 10, it can be seen from the bar chart of “P” and “PN” that the mismatching elimination step can effectively improve the RR of our homing method. There are 15 different goal positions tested; compared to the warping method, our method performs better at 13 goal positions. From Figure 10a,b, although the RR for the two methods drops slightly with the changing of a tall plant in the experiment scene, our homing method still performs better. The performance for the two methods is greatly influenced when the illumination changes in the experiment scene, as shown in Figure 10a,c. Especially for the warping method, the homing performance declines dramatically. The results of RR turn out to be the same as those of AHC: compared to the warping method, our homing method has better robustness to the changes of the environment in the scene.

### 4.5. Homing Trials in a Real Scene

**Figure 11:**
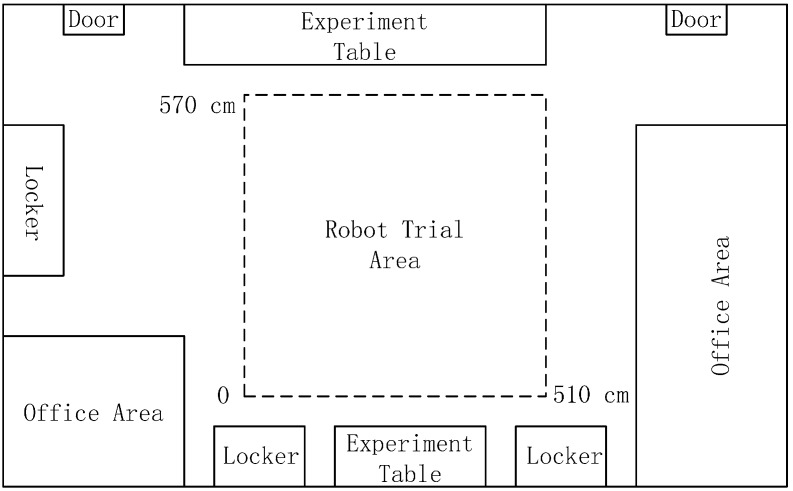
Robot trial environment in the real scene.

In order to further evaluate the performance of our method in practice, experiments were conducted in a real scene. We selected the intelligent robot lab of Harbin Engineering University as the experiment scene. The surroundings of the trial area are shown in Figure 11. The tracked mobile robot introduced in Section 4.1 was used in the trials. We randomly chose four goal positions, which were uniformly distributed in the trial area. Five current positions for each goal position spaced evenly throughout the area were selected for tests. The robot took the panoramic image at its current position, compared it to the goal image stored in memory and computed the homing angles separately by the proposed method and the warping method. After that, in the homing direction, the robot moved a step at the fixed length of 25 cm. In the trials, the above process would repeat until the robot reached a place within the range of 30 cm of the goal position or its movement distance was more than half of the circumference of the trial area, which was when the robot moves no more than 43 steps. If the robot can arrive at the goal position within the prescribed steps, the homing is successful; otherwise, the homing has failed. Because most stopping criteria based on image information are likely to lead the robot to oscillate around the goal position, the robot was manually stopped once it reached the goal area.

**Figure 12:**
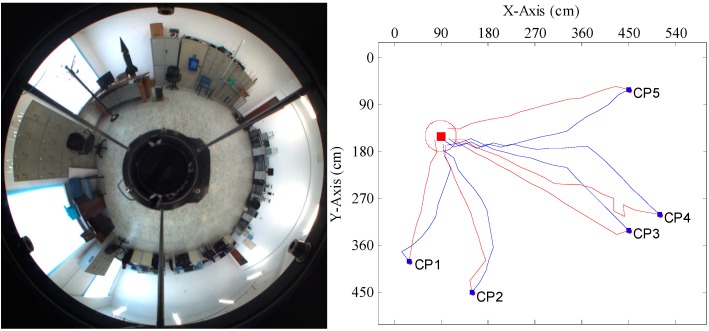
Robot homing Trial 1. Top left: the panorama of the goal position; top right: the homing trajectories for five different current positions; the table below: the number of homing steps and the average angular error for each current position. CP, current position.

**Figure 13:**
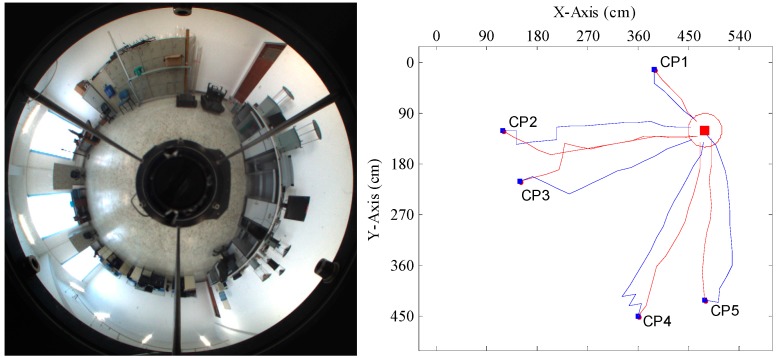
Robot homing Trial 2.

**Figure 14:**
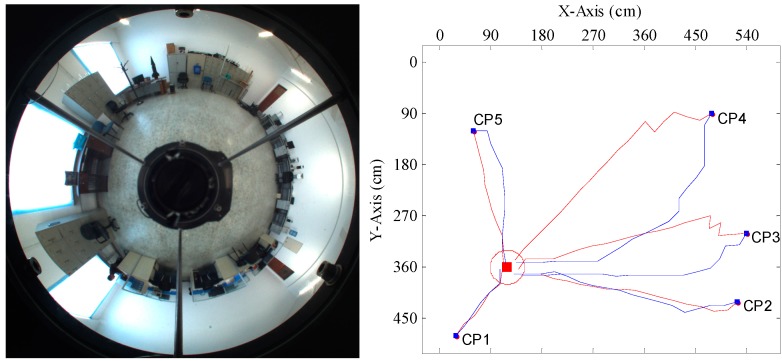
Robot homing Trial 3.

**Figure 15:**
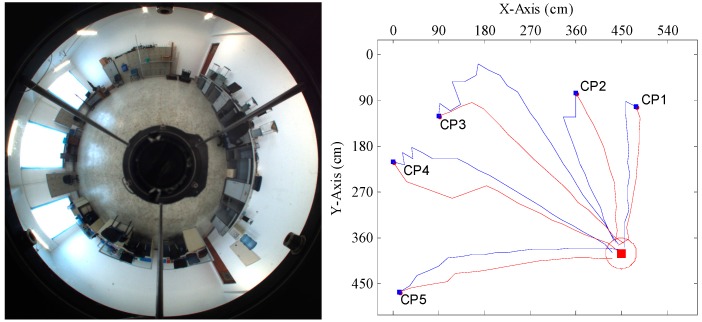
Robot homing Trial 4.

Figure 12, Figure 13, Figure 14 and Figure 15 show the trajectories of the robot for the two methods, with tables listing the statistics of the number of homing steps *N* and the average angular errors *σ*(°) in each trial. The goal area is indicated by the red circle. “CP” represents the current position. Red lines represent the trajectories of the proposed method. Blue lines represent those of the warping method. As shown in Figure 12, Figure 13, Figure 14 and Figure 15, most homing trajectories for the warping method are more curved than those for our method. In total, 20 homing trials were carried out, 17 of which suggest that the average angular errors for our method are smaller; the number of total homing steps for the warping method is 312, while for our method, that number is only 292. Both the trajectories and statistics indicate that the homing angles computed by our method are more accurate, and the movement distance is shorter.

**Table:** 

Method	CP1		CP2		CP3		CP4		CP5	
	*N*	*σ*	*N*	*σ*	*N*	*σ*	*N*	*σ*	*N*	*σ*
Warping	11	24.54	13	21.12	17	15.03	20	19.14	16	18.78
Proposed	10	6.34	13	11.57	16	5.81	20	15.43	15	6.47

**Table:** 

Method	CP1		CP2		CP3		CP4		CP5	
	*N*	*σ*	*N*	*σ*	*N*	*σ*	*N*	*σ*	*N*	*σ*
Warping	5	9.66	16	15.23	14	11.79	17	19.09	14	20.19
Proposed	5	6.47	15	11.42	15	14.92	14	9.29	12	8.31

**Table:** 

Method	CP1		CP2		CP3		CP4		CP5	
	*N*	*σ*	*N*	*σ*	*N*	*σ*	*N*	*σ*	*N*	*σ*
Warping	6	8.01	17	10.65	18	14.79	21	23.82	11	13.89
Proposed	6	8.13	17	8.07	20	16.24	20	11.89	10	5.10

**Table:** 

Method	CP1		CP2		CP3		CP4		CP5	
	*N*	*σ*	*N*	*σ*	*N*	*σ*	*N*	*σ*	*N*	*σ*
Warping	13	15.13	14	12.93	27	31.89	23	15.99	19	12.14
Proposed	12	9.50	13	9.89	20	15.22	21	11.32	18	5.29

## 5. Conclusions

This paper proposes a novel method to solve the problem of local visual homing. The method is composed of a novel visual homing algorithm and a novel mismatching elimination algorithm. The former is inspired by the warping method, and the latter is based on the distribution characteristics of landmarks in the initial panoramic image. Compared to the warping method, the proposed homing method improves the homing accuracy effectively and has better robustness to the changes in the environment. Experiments on image databases and in a real scene confirm the improved performance.

For the visual homing algorithms based on landmarks, a reduction in the number of landmarks can effectively reduce the amount of computation, while the homing performance might be affected. In the future, we will focus on how to reduce the number of landmarks effectively on the premise of guaranteeing the homing precision.
